# Moderators of an intervention on emotional and behavioural problems: household- and school-level parental education

**DOI:** 10.1093/eurpub/ckac143

**Published:** 2022-10-18

**Authors:** Nil Horoz, J Marieke Buil, Susanne Koot, Frank J van Lenthe, Tanja A J Houweling, Joost Oude Groeniger, Hans M Koot, Pol A C van Lier

**Affiliations:** Department of Clinical, Neuro- and Developmental Psychology, Vrije Universiteit Amsterdam, Amsterdam, The Netherlands; Amsterdam Public Health Research Institute, Amsterdam, The Netherlands; Department of Clinical, Neuro- and Developmental Psychology, Vrije Universiteit Amsterdam, Amsterdam, The Netherlands; Amsterdam Public Health Research Institute, Amsterdam, The Netherlands; Research Centre Urban Talent, Rotterdam University of Applied Sciences, Rotterdam, The Netherlands; Radboud University Medical Center, Nijmegen, The Netherlands; Department of Public Health, Erasmus MC, University Medical Center Rotterdam, Rotterdam, The Netherlands; Department of Human Geography and Spatial Planning, Faculty of Geosciences, Utrecht University, Utrecht, The Netherlands; Department of Public Health, Erasmus MC, University Medical Center Rotterdam, Rotterdam, The Netherlands; Department of Public Health, Erasmus MC, University Medical Center Rotterdam, Rotterdam, The Netherlands; Department of Public Administration and Sociology, Erasmus University Rotterdam, Rotterdam, The Netherlands; Department of Clinical, Neuro- and Developmental Psychology, Vrije Universiteit Amsterdam, Amsterdam, The Netherlands; Amsterdam Public Health Research Institute, Amsterdam, The Netherlands; Department of Clinical, Neuro- and Developmental Psychology, Vrije Universiteit Amsterdam, Amsterdam, The Netherlands; Amsterdam Public Health Research Institute, Amsterdam, The Netherlands

## Abstract

**Background:**

Children of lower-educated parents and children in schools with a relatively high percentage of peers with lower-educated parents (lower parental education schools) are more likely to develop emotional and behavioural problems compared to children in higher-educated households and schools. Universal school-based preventive interventions, such as the Good Behaviour Game (GBG), are generally effective in preventing the development of emotional and behavioural problems, but information about potential moderators is limited. This study examined whether the effectiveness of the GBG in preventing emotional and behavioural problems differs between children in lower- and higher-educated households and schools.

**Methods:**

Using a longitudinal multi-level randomized controlled trial design, 731 children (*M*_age_=6.02 towards the end of kindergarten) from 31 mainstream schools (intervention arm: 21 schools, 484 children; control arm: 10 schools, 247 children) were followed annually from kindergarten to second grade (2004–2006). The GBG was implemented in first and second grades.

**Results:**

Overall, the GBG prevented the development of emotional and behavioural problems. However, for emotional problems, the GBG-effect was slightly more pronounced in higher parental education schools than in lower parental education schools (*B*_higher parental education schools_ =−0.281, *P* <0.001; *B*_lower parental education schools_ =−0.140, *P* *=* 0.016). No moderation by household-level parental education was found.

**Conclusions:**

Studies into universal school-based preventive interventions, and in particular the GBG, should consider and incorporate school-level factors when studying the effectiveness of such interventions. More attention should be directed towards factors that may influence universal prevention effectiveness, particularly in lower parental education schools.

## Introduction

Poor mental health among school-aged children, including emotional and behavioural problems, is a global public health concern.^1^ Without intervention, emotional and behavioural problems that develop during elementary school have been shown to increase the risk of many concurrent and future negative outcomes, such as mental disorders, physical health problems, academic failure, criminality and unemployment in adulthood.^1–3^ Mental health problems cause a large proportion of the global disease burden and are estimated to account for 32.4% of years lived with disability and 13% of disability adjusted life years.^4^ Therefore, early prevention of emotional and behavioural problems is an urgent matter. Elementary schools are accessible and practical settings for the implementation of preventive (universal) interventions. Universal school-based preventive interventions (i.e. those delivered to all children) may be key to effective preventive efforts. One such programme is the Good Behaviour Game (GBG),^5^ which has been proven effective in preventing the development of children’s behavioural and emotional problems.^6–9^

The GBG has previously been referred to as a ‘behavioural vaccine’ due to its cost-effectiveness and its ability to prevent mental health problems across diverse cultures and populations.^6^ It aims to prevent mental health problems in healthy children and in children at risk of developing mental health problems. When implemented on a large scale in early primary education, universal school-based interventions like the GBG have the capacity to reach large quantities of broad populations, including children who may be otherwise hard to reach. However, in more recent research, it has been shown that the GBG may differentially affect children with varying risk profiles and that its benefit may not equally extend to children with higher family-demographic risk profiles.^10^ This challenges the notion that the GBG is a ‘behavioural vaccine’ and should be further explored. Thus, we investigate whether the effect of the GBG is moderated by a well-established risk factor at both the household and school levels.

Across nations, a robust risk factor of poor child mental health at both the household and school levels is low socioeconomic status (SES).^11^ In the Netherlands, where this study was conducted, school-level socioeconomic inequalities within and between schools are measured by children’s parents’ education levels.^12^ Children of lower-educated parents (and higher-educated parents) are likely to attend schools with children from similar parental education backgrounds.^13^ Already in elementary school, children of lower-educated parents and children in schools with a high percentage of students with lower-educated parents (lower parental education schools) are at a higher risk of developing emotional and behavioural problems.^14^ This may be due to the risk factors that are associated with lower-educated households (e.g. less resources at home and less cultivating parenting strategies) and with lower parental education schools (e.g. less effective school management and more teacher distress).^15^^,^^16^ On the one hand, interventions like the GBG may have the potential to decrease inequalities in the prevalence of mental health problems in children from lower- and higher-educated contexts. On the other, they may be less effective in decreasing inequalities owing to factors related to lower household- and school-level parental education because these factors may reduce the effectiveness of the intervention. However, it remains unknown whether the impact of the GBG indeed differs between children from lower- and higher-educated households and schools.

The majority of the school-based intervention studies on children’s emotional and behavioural problems have not included household- or school-level parental education or only included SES as a descriptive or a study variable.^17^^,^^18^ Some of these studies examined either children from low SES households^10^^,^^19^ or low SES schools alone^19–23^ and thereby lack a comparison group. Additionally, studies that did use SES as a moderator did not account for SES at both the household and school levels.^17^^,^^18^^,^^24–28^ Not accounting for SES at both levels may lead to the misleading conclusion that the effects are explained solely by either household- or school-level SES.^14^ Therefore, this study provides a novel approach by allowing a more detailed examination of the moderating role of a well-established risk factor at both levels. Specifically, we examine whether household- and school-level parental education moderate the effectiveness of the GBG in preventing the development of Dutch children’s emotional and behavioural problems from kindergarten to second grade.

## Methods

### Sample

Participants were recruited from the first 31 elementary schools in rural and urban areas of the Netherlands that agreed to participate in the research project. Schools could participate if they were willing to implement the GBG (if randomly selected in the intervention arm) or if they were willing to be on a waiting list (if randomly selected in the control arm).

**Figure 1:**
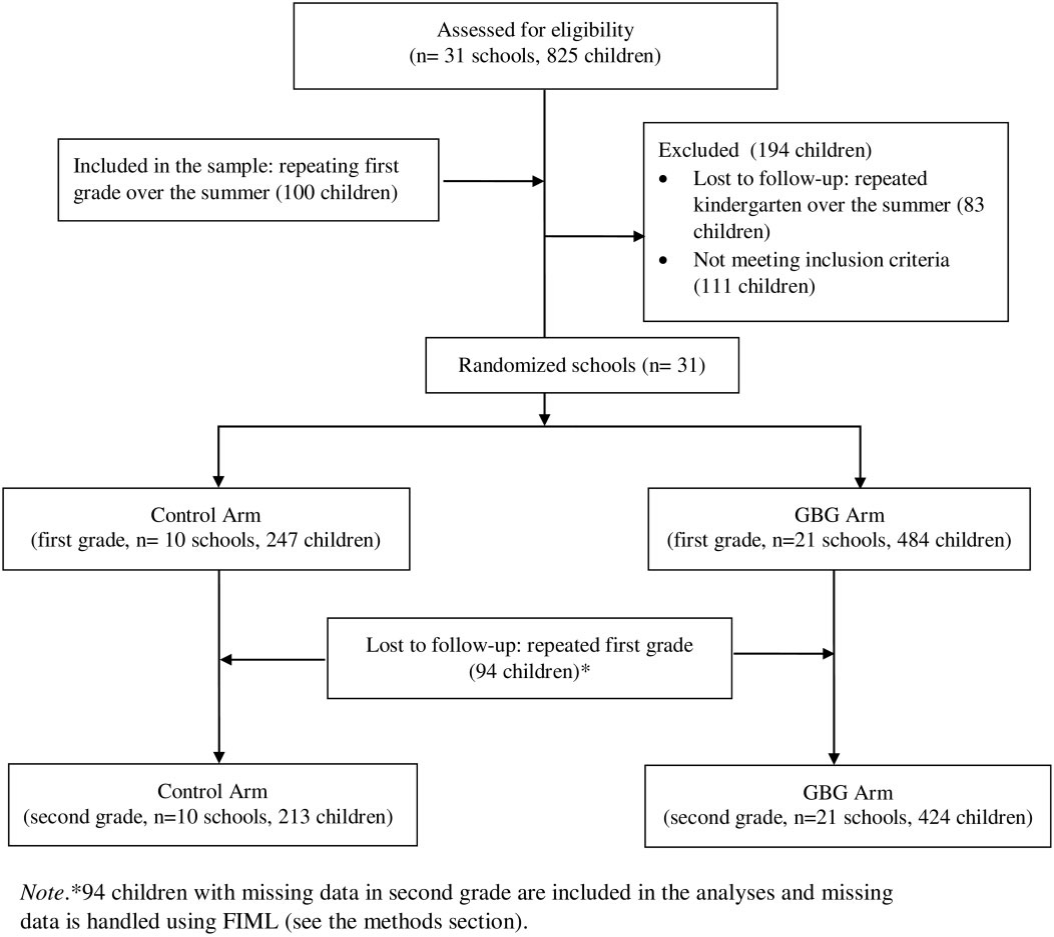
Flowchart of the cluster randomized participants included in the randomized control trial, adapted with permission from Witvliet and colleagues^30^

Children’s emotional and behavioural problems were annually assessed for 3 years, from kindergarten (*M*_age_=6.02, SD= 0.46) to second grade (in spring). Inclusion criteria were (i) active parental consent, (ii) data on school-level parental education and (iii) at least two out of three completed waves of teacher-reported data on emotional and behavioural problems. In total, out of 825 children who were initially included in the study, 731 (50% girls) fulfilled these criteria (see the flowchart in figure 1). All children had complete data on school-level parental education, 18.5% had missing data on household-level parental education and 24% had missing data on emotional and behavioural problems for one wave.

### Design and procedure

Participating schools were randomly assigned, with an oversampling of intervention schools, to either the control (10 schools, *n* = 247 children) or the GBG intervention arm (21 schools, *n* = 484 children). See Supplementary appendix A for sample size determination. The first assessments of emotional and behavioural problems were conducted in the Spring of 2004 when participants were in kindergarten (pre-intervention). In first and second grades, the GBG intervention was implemented and the second and third assessments were conducted.

### The GBG

The GBG is a classroom-based preventive intervention that aims to prevent disruptive behaviour by creating a positive and a predictable classroom environment where children work in teams and stimulate each other to show appropriate classroom behaviour. The GBG is implemented in classrooms by teachers for 15–60-min periods while students are working on regular school tasks. Before the GBG period, teachers and students select positively formulated classroom rules. Teachers then identify and assign children to teams of 4–5 students with an equal number of disruptive and non-disruptive children and give each team a set of cards. During the game, if a team member violates one of the preselected rules, teachers take a card from that team. Teams are rewarded at the end of the game period if at least one card remains. Teachers praise teams and children by complimenting appropriate behaviour and, aside from removing cards from teams that violate the rules, do not pay attention to disruptive behaviour. The GBG is implemented in three phases: introduction, expansion and generalization. In the introduction phase, the GBG is played three times a week. In the expansion and generalization phases, the duration (hours/days) is extended. More information regarding the intervention strategy, implementation and teacher training is described elsewhere.^29^

### Measures

*Household-level parental education* was based on the highest education level per household, obtained by the (two) parent/caregiver(s). Parental education levels were ranked according to the Dutch Standard Education Classification,^30^ which corresponds to the International Standard Classification of Education (ISCED).^31^ Following the ISCED classifications, parental education levels were coded using an 8-point scale, with education levels ranging from 0= no education/early education, 1= primary education, 2= lower secondary education, 3= upper secondary education, 4= post-secondary non-tertiary education, 5= short-cycle tertiary education, 6 =bachelor’s degree or equivalent, to 7 =master’s degree, equivalent or higher. The household parental education levels were reverse coded so that higher scores indicated lower parental education levels.

*School-level parental education* levels were determined by the per-school percentage of children of low-educated parents. In the Netherlands, school-level socioeconomic inequalities are measured by children’s parents’ education levels. The Netherlands Inspectorate of Education calculates the percentage of low parental education levels of each school to identify schools that qualify for additional governmental resources.^12^ Low education is defined as parent(s) completing no more than elementary school. Thus, in this study, school-level parental education was based on the percentage score of low parental education levels of the entire school population. The percentage scores can range from 0% to 100%, with higher percentage scores indicating schools with higher percentages of children of low-educated parents. This information is publicly available (www.duo.nl).

*Teacher ratings of individual children’s behavioural and emotional problems* were assessed by the Problem Behaviour at School Interview (PBSI).^32^ The PBSI is a validated questionnaire conducted via interview that uses a 5-point Likert scale ranging from 0 (never applicable) to 4 (often applicable).^33^ Behavioural problem scores were assessed by conduct problems (12 items) and oppositional defiant problems (7 items), and calculated as the average of the mean scores of the two subscales. Emotional problem scores were assessed by depression (7 items) and anxiety (5 items) symptoms, and the same procedure was followed. Higher scores indicated higher levels of emotional and behavioural problems. See Supplementary appendix A for more information regarding the PBSI and the outcome variables.

*Intervention status* was dummy-coded (0 =control, 1 = GBG).

*Covariates* included gender (0 =girls, 1 =boys) and cluster size. Cluster size (i.e. number of participating children per school) was grand-mean centred and included to account for unequal cluster sizes (*M*=23, range =8–88; mode =14, median =20). Baseline differences in kindergarten were controlled for because—despite randomization—children in the GBG arm had moderately higher levels of emotional [*M*_GBG_=0.85, SD=0.57; *M*_control_=0.67, SD=0.55, *t*(647)=−3.85, *P*<0.001, Cohen’s *d*=0.32] and slightly higher levels of behavioural problems [*M*_GBG_=0.80, SD=0.67; *M*_control_=0.69, SD=0.65, *t*(650)=−2.08, *P*=0.038, Cohen’s *d*=0.17] than children in the control arm.

### Statistical analyses

A parallel latent growth curve (LGM) model with two-level time-nested-within-individual data structure (1 =variation across individual children, 2 =variation across schools), in which the development of emotional and behavioural problems was conceptualized by latent growth parameters (intercept and a linear slope), was used to test the main effects and potential moderation by household- and school-level parental education of the GBG in preventing the development of emotional and behavioural problems. The intercept represented the initial level in kindergarten (baseline) and the slope represented change over time (from kindergarten to second grade).

The analyses were conducted in three steps. All models were fitted in Mplus version 8.0.^34^ We first computed design effects. Design effects larger than 2.0 indicate significant clustering of the data at the school level [Design effects =1+(*n*_c_−1)ICC].^35^ In the second step, we tested for main effects of the GBG intervention by regressing the outcome on the GBG intervention status, adjusting for the baseline differences in emotional and behavioural problems. In the third step, we tested moderation by household- and school-level parental education via a cross-level interaction and a between(school)-level interaction, respectively. Before examining cross-level interactions between household-level parental education and the GBG, we checked whether such interactions could be performed. To do this, we modelled a random slope at the (within)household-level and estimated its variance at the (between)school-level. This random slope represented the effect of household-level parental education on the growth parameters of children’s (individual-level) emotional or behavioural problems. Then, using Satorra Bentler chi-square difference tests, we checked whether adding a random slope improved the model fit of the main effect model in Step 2. If this was the case, the random slope parameter was regressed on the GBG at the between-level (i.e. cross-level interaction) to test the interaction between household-level parental education and the GBG on the development of individual-level emotional and behavioural problems. To test for moderation by school-level parental education at the between-level, an interaction term between school-level parental education and the GBG was added as a predictor of between-level emotional and behavioural problem development.

Model fit indices for multi-level latent growth models were used to determine model fit at both the household and school levels. For specifics, see Supplementary appendix Btable S1. MLR estimators (maximum likelihood estimation with robust standard errors) were used to account for the possible non-normal distribution of data. Missing data were therefore handled using the default option in Mplus for MLR-estimation with missing at random data (i.e. Full Information Maximum Likelihood estimation). To ensure that the results were robust, two additional sensitivity tests were done: (i) by imputing the missing data in MPLUS (*N* = 25 imputed datasets) and (ii) by testing the models on a subsample (*N*=596) with complete household-level parental education data.

### Ethics

This study was approved by the Medical Ethics Committee of the Vrije Universiteit Amsterdam Medical Center and was registered with the ‘Netherlands Trial Register’ [Trial NL470 (NTR512)] (www.trialregister.nl). Signed parental informed consent was obtained from parents. Parents and children could revoke participation at any time.

## Results

### Descriptive statistics

Descriptive statistics of household- and school-level parental education of the whole sample are presented in table 1. The household-level parental education levels were slightly higher in the control arm than in the GBG arm, *t*(1)=2.75, *P*=0.006, Cohen’s *d*=0.24.

**Table 1:** Descriptive statistics of household- and school-level parental education of the whole sample

Household-level parental education (*N *=* *731)	*N* (%)	Low school-level parental education (*N *=* *31)	%
No education/early education	11 (1.5)	Range	0.0–76.5
Primary education	43 (5.9)	Mean	16.4
Lower secondary education	57 (7.8)	Standard deviation	19.2
Upper secondary education	72 (9.8)	Mode	7.3
Post-secondary non-tertiary education	46 (6.3)	Median	8.1
Short-cycle tertiary education	149 (20.4)		
Bachelor’s or equivalent degree	124 (17.0)		
Master’s or equivalent degree	94 (12.9)		
Missing	135 (18.5)		

The per-school percentage of children of low-educated parents was not significantly different between the schools in the control (*M*=18.61%, SD=23.97%) and intervention arms (*M*=15.35%, SD=17.02%), *t*(29)=0.44, *P*=0.666, Cohen’s *d*=0.17. The correlation between household- and school-level parental education in our sample was positive and of moderate magnitude (*r*=0.42, *P*<0.001).

### Model building, unconditional latent growth models per condition and the GBG main effects

Intra-class correlations, design effect values, model fit indices of the unconditional LGMs for the whole sample and model building testing results are presented in Supplementary appendix Btable S1. Design effects indicated the need to use a two-level structure to analyze the data. Model fit indices were acceptable for both outcomes. Adding the random slope improved the model fit of the main effect model of emotional problems only, which indicated that cross-level interaction testing can be performed for emotional but not for behavioural problems.

Results from the unconditional LGMs (Supplementary appendix Btable S2) showed that in the GBG arm emotional and behavioural problems stayed stable over time, as indicated by the non-significant slopes (emotional problems: *B*=0.065, *P*=0.115; behavioural problems: *B*=−0.041, *P*=0.177). In the control arm, there was a significant yearly increase of emotional problems (*B *=* *0.271, *P* < 0.001) and a borderline significant yearly increase of behavioural problems (*B*=0.100, *P*=0.057). This indicates that without the GBG, emotional (and to a lesser extent behavioural) problems tended to increase from kindergarten to second grade.

Results of main effects (table 2) showed that the GBG was effective in preventing the increase in emotional problems that was found in the control group [*B*=−0.208, 95% CI (−0.345, −0.070), *P* *=* 0.003]. In addition, the GBG was also effective in preventing behavioural problems from kindergarten to second grade [*B*=−0.133, 95% CI (−0.256, −0.010), *P*=0.034].

**Table 2:** Main effects of the GBG and moderation by household- and school-level parental education on children’s emotional and behavioural problems

	Emotional problems								Behavioural problems							
	Intercept				Slope				Intercept				Slope			
Main effects model	*B*	SE	95% CI	*β*	*B*	SE	95% CI	*β*	*B*	SE	95% CI	*β*	*B*	SE	95% CI	*β*
Within level																
Gender	0.054	0.033	−0.011, 0.120	0.189	−0.033	0.032	−0.095, 0.029	−0.242	0.361	0.041	0.281, 0.441***	0.746	−0.032	0.024	−0.080, 0.015	−1.51
Lower parental education	0.013	0.011	−0.010, 0.035	0.086	0.008	0.009	−0.010, 0.026	0.113	0.057	0.017	0.024, 0.091***	0.228	−0.007	0.007	−0.021, 0.006	−0.657
Between level																
Cluster size	0.002	0.002	−0.002, 0.005	0.107	−0.004	0.002	−0.008, 0.000	−0.286	0.001	0.002	−0.002, 0.004	0.074	−0.002	0.001	−0.005, 0.000	−0.257
School-level parental education	0.002	0.002	−0.002, 0.006	0.187	−0.002	0.002	−0.007, 0.002	−0.215	0.005	0.002	0.001, 0.010*	0.419	0.001	0.001	−0.002, 0.004	0.113
GBG	0.161	0.088	−0.010, 0.333	0.740	−0.208	0.070	−0.345, −0.070**	−1.05	0.140	0.087	−0.031, 0.310	0.604	−0.133	0.063	−0.256, −0.010*	−0.964
Interaction effect model																
Cluster size	0.002	0.002	−0.002, 0.005	0.107	−0.004	0.002	−0.007, 0.000*	−0.270	0.001	0.002	−0.002, 0.004	0.074	−0.002	0.001	−0.004, 0.000*	−0.252
School-level parental education	0.002	0.002	−0.002, 0.006	0.189	−0.006	0.002	−0.010,−0.003***	−0.588	0.005	0.002	0.001, 0.010*	0.420	0.000	0.002	−0.004, 0.003	−0.055
GBG	0.162	0.088	−0.011, 0.335	0.737	−0.211	0.066	−0.339, −0.082***	−1.06	0.140	0.087	−0.030, 0.310	0.604	−0.134	0.063	−0.257, −0.012*	−0.984
Household-level parental education—× GBG		–	–	–	0.010	0.033	−0.055, 0.074	n.a.	–	–	–	–	–	–	–	–
School-level parental education × GBG	–	–	–	–	0.007	0.003	0.002, 0.013**	0.510	–	–	–	–	0.002	0.003	−0.003, 0.007	0.231

*Note*: *P < .05, **P < .01, ***P < .001; n.a. = estimate not available due to the use of Monte Carlo integration to estimate cross-level interaction. Note that the effect of school-level parental education is small because it represents the effect at 1% change in school-level parental education.

### Moderation by household- and school-level parental education of the GBG impact

*Household level*: Results showed no significant cross-level interaction between household-level parental education and the GBG-effect on individual-level emotional problem development, *B*=0.010, 95% CI (−0.055, 0.074), *P*=0.765 (see table 2). The cross-level interaction for behavioural problems was not tested.

**Figure 2:**
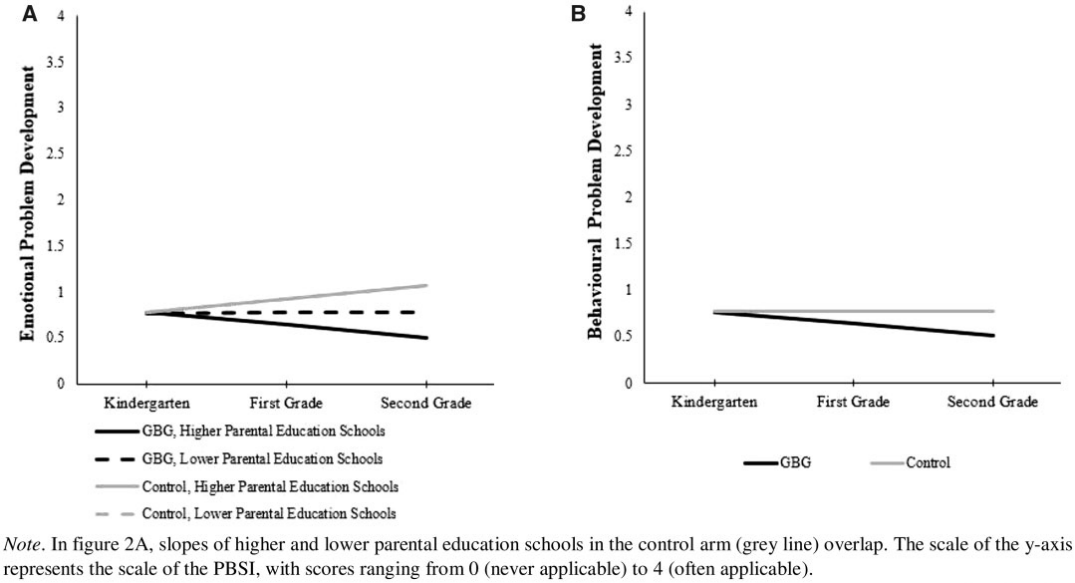
School-level parental education effects on the development of emotional problems (A) and behavioural problems (B) in GBG vs. control arms

*School level:* Results showed a significant interaction between school-level parental education and the GBG-effect on children’s emotional problems, *B*=0.007, 95% CI (0.002, 0.013), *P* *=* 0.005 (see table 2). That is, the GBG was more effective in preventing the development of emotional problems in higher parental education schools than in lower parental education schools. Figure 2A shows a visual representation of this interaction effect in which the effects were probed at 0.50 SD above [lower parental education schools; ∼26% of the total sample; *B*=−0.140, SE=0.059, 95% CI (−0.255, −0.026), *P* *=* 0.016] and at 0.50 SD below the mean of school-level parental education [higher parental education schools; ∼7% of the total sample; *B*=−0.281, SE=0.080, 95% CI (−0.438, −0.124), *P* *<* 0.001]. For behavioural problems, no moderation between school-level parental education and the GBG was found, *B*=0.002, 95% CI (−0.003, 0.007), *P* = 0.382 (see figure 2B). The two sensitivity tests showed no changes in interpretation of the results. For specifics, see Supplementary appendix Btables S3 and S4.

## Discussion

Overall, the GBG prevented the development of emotional and behavioural problems from kindergarten to second grade. Specifically, results showed that the effectiveness of the GBG in preventing emotional and behavioural problems did not differ between children of lower- and higher-educated parents. Nevertheless, the GBG was more effective in schools with a lower (compared to higher) percentage of children of lower-educated parents, albeit only for emotional problems.

To our knowledge, this study provides preliminary evidence that school-level parental education may impact the effectiveness of the GBG in reducing emotional problems. Previous studies mainly tested household/individual-level factors, such as gender, initial risk status and behaviour type as moderators of universal school-based programmes like the GBG.^36^^,^^37^ This study suggests that more attention needs to be directed towards lower parental education schools and that in addition to individual-level moderators, school-level moderators should be studied to better understand the potential differential impact of universal school-based interventions.

The characteristics of lower and higher parental education schools may explain why the GBG was less effective in lower parental education schools for emotional problems. Lower parental education schools may have fewer resources, less effective school management, less teacher support and teachers who are insufficiently prepared to deal with such schools’ diverse populations.^16^^,^^38^ Nevertheless, this study cannot explain why the school-level interaction effect was found for emotional but not for behavioural problems. It stands to reason that the GBG is more directed towards behavioural problems. Thus, it may be less affected by possible school-level factors that may attenuate its impact. However, we should be cautious in interpreting the results before replication studies with longer follow-up procedures are conducted.

The following limitations should be noted. First, and most importantly, we did not have implementation fidelity data. It is possible that there were no major differences in implementation fidelity based on school-level parental education since the interaction effect between school-level parental education and the GBG on behavioural and emotional problems differed. Our study should be considered as an effectiveness trial and an exploratory study meant to stimulate further investigation. It is important to study, for instance, whether the GBG’s weaker effect on emotional problems in lower parental education schools is due to (i) specific school-level factors, (ii) possible problems with implementation or (iii) to lack of components more directly targeting emotional problems. Second, our sample was not randomly drawn from the Dutch population of elementary schools. Third, we used teacher-reports and teachers were not blinded to condition. Self-reports and observational data, which could have provided additional insights, were not available. Fourth, sample size at the between-level was relatively small with 31 schools. Due to this we did not, for example, have enough power to test a three-way interaction of the GBG, household- and school-level parental education. Finally, we used parental education as an index of broader SES. Future replication studies are encouraged to use broader SES indices.

Despite these limitations, our results have implications for research and practice. Testing implementation fidelity and school-level moderators that relate to lower parental education schools would result in determining the specific factors to be addressed, such as teacher support and training or implementation infrastructure in schools. Furthermore, if lower parental education schools need more support in preventing emotional problems, more intensified or selective interventions that target high-risk populations could be implemented in these schools. Nevertheless, it is noteworthy that for general prevention efforts the GBG was equally effective in preventing behavioural and emotional problems irrespective of household-level parental education and in preventing behavioural problems irrespective of school-level parental education. Although results suggested that the GBG was less effective in lower parental education schools, it still was an effective tool for preventing the development of emotional problems in these schools. School-based universal interventions reduce the potential that children who may be at risk of developing mental health problems or who may be otherwise difficult to reach will be overlooked. For instance, despite the need for mental health services, it has been shown that the majority of low SES children do not receive treatment.^39^ At a time in which SES-related inequalities are on the rise,^40^ this study shows that the GBG is effective in preventing the development of behavioural and emotional problems of children in lower- and higher-educated households and schools while suggesting that more attention should be directed towards lower parental education schools.

## Supplementary data

Supplementary data are available at *EURPUB* online.

## Funding

This study was supported by a grant from the Netherlands Organization for Health Research and Development (ZonMw) (project No. 531003013) and by ZonMw Grants #26200002 and #120620029.T.A.J.H. was funded through a grant awarded by the Norwegian Research Council (project number 288638) to the Centre for Global Health Inequalities Research (CHAIN) at the Norwegian University for Science and Technology (NTNU).

*Conflicts of interest*: None declared.

Key points

- The effectiveness of the GBG in preventing the development of behavioural and emotional problems did not differ between children of lower- and higher-educated parents from kindergarten to second grade.
- The GBG is equally effective in preventing the development of behavioural problems in schools with higher and lower percentages of children with lower-educated parents, but less effective in preventing the development of emotional problems in lower parental education schools than in higher parental education schools.
- When testing intervention effectiveness, school-level variables as moderators should be included in study designs.
- More attention should be directed towards schools with a higher percentage of children with lower-educated parents.

## Supplementary Material

ckac143_Supplementary_Data

## Data Availability

The data underlying this article will be shared upon reasonable request to the corresponding author. Mplus code is available via the link: https://osf.io/pxs3w/?view_only=bd0cbd3813b84805bbeb0339e108ac41.
