# Supplementary material for: Moderators of an intervention on emotional and behavioural problems: household- and school-level parental education
Source: Eur J Public Health. 2022 Oct 18;32(6):864–70. doi: 10.1093/eurpub/ckac143 (PMC9713443; doi:10.1093/eurpub/ckac143)
Supplement: ckac143_Supplementary_Data [file ckac143_supplementary_data.docx]

**Appendix A**

**Supplementary Method**

**Sample Size Determination**

With power of 0.80 and a conventional alpha of 0.05, at least 14 clusters were needed to detect differences considered relevant for clinical practice with an effect size of 0.50 (which is higher than the recommended minimum effect size of 0.41(1)) between the intervention and control arms and at least 28 clusters were needed to test for moderation effects when using a dichotomous moderator (e.g., gender). 31 schools that expressed a wish to participate in the research study were included.

**Problem Behaviour in School Interview (PBSI)**

Teacher ratings of individual *children’s behavioural and emotional problems* were assessed by the Problem Behaviour at School Interview (PBSI)(2). The PBSI was administered by trained research assistants. Via a structural interview, trained research assistants asked teachers to rate children’s emotional and behavioural problems using a 5-point Likert scale ranging from 0 (never applicable) to 4 (often applicable). Higher scores indicated higher levels of behavioural and emotional problems.

Behavioural problem scores were calculated as the average of the mean scores of the subscales of conduct problems and oppositional defiant problems. *Conduct problems* were assessed by 12 items: “threatens other people”, “starts fights”, “pushes or endangers other children”, “bullies or is mean to others”, “physically attacks others”, “destroys someone else’s property”, “tells lies”, “swears or uses bad language”, “truant or absent without a valid reason”, “steals”, “hangs out with deviant friends”, “does not feel guilty if misbehaves.” The Cronbach’s alphas ranged from 0.885 to 0.918 from kindergarten to second grade. *Oppositional defiant problems* were assessed by 7 items: “rebellious”, “stubborn”, “does not adhere to school rules”, “disobedient”, “has tantrums or easily loses their temper”, “talks back”, “argues”. The Cronbach’s alphas ranged from 0.890 to 0.905 across the three years. The correlation between the two subscales of behavioural problems ranged from 0.83 - 0.84 across grades.

Emotional problem scores were calculated as the average of the mean scores of the subscales of depression symptoms and anxiety symptoms. *Depression symptoms* were assessed by 7 items: “unhappy or depressed”, “doesn’t like or enjoy many things”, “indifferent, listless or unmotivated”, “cries or is sad at school”, “burdened by feelings of guilt”, “lack of energy”, “feels inferior”. The Cronbach’s alphas ranged from 0.778 to 0.826 from kindergarten to second grade. *Anxiety symptoms* were assessed by 5 items: “worries about many things”, “anxious”, “nervous or tense”, “too dependent on adults”, “afraid of going to school.” The Cronbach’s alphas ranged from 0.813 to 0.836 across the three years. The correlation between the two subscales of emotional problems ranged from 0.66 - 0.69 across grades.

The convergent validity of the PBSI was tested in a sample that comes from the same research project as our study’s sample by estimating the correlations between the behavioural and emotional scales of the PBSI and the Teacher’s Report Form(3, 4). The correlations for behavioural problems were .75 (*p* < .01) and were 0.55 for emotional problems (*p* < .01)(4). Furthermore, measurement invariance of teacher ratings of emotional and behavioural problems between lower- and higher-educated households and schools has been established elsewhere(5).

The Kolmogorov-Smirnov and Shapiro-Wilk tests showed that emotional and behavioural problems were not normally distributed (*p* < .001). However, the values of skewness (emotional problems range: 0.535 – 0.663; behavioural problems range: 1.051 – 1.053) and kurtosis (emotional problems range: -0.153 – 0.315; behavioural problems range: 0.652 – 0.769) were within the critical bounds.

Our sample is considered a convenience sample drawn from the general population. Thus, since our participants come from non-clinical populations, we do not expect average higher scores in the PBSI. The scale of the PBSI range from 0 – 4, with higher scores indicating more problems. The mean scores of behavioral problems for the whole sample were 0.76 in kindergarten (*SD* = 0.67, *range* = 0 – 3.14), 0.74 (*SD* = 0.66, *range* = 0 – 3.17 ) in first grade and 0.70 (*SD* = 0.67, *range* = 0- 3.36) in second grade. The mean scores of emotional problems for the whole sample were 0.78 (*SD* = 0.57, *range* = 0 – 3.21) in kindergarten, 0.88 (*SD* = 0.61, *range* = 0 – 3.05) in first grade and 0.97 (*SD* = .72, *range* = 0 – 3.38) in second grade. Thus, the mean scores of emotional and behavioral problems were in the lower range of the PBSI scale.

Furthermore, we calculated the means of behavioural and emotional problems across three years for children in lower-educated households and schools as well as for children in higher-educated households and schools. Lower and higher household- and school-level parental education were calculated by 0.50 SD above and below the mean score of household- and school-level parental education.

Compared to children in higher parental education schools (< 7% of low educated parents per school), children in lower parental education schools ( > 26% of low educated parents per school) had significantly higher levels of behavioural problems across the three years and significantly higher levels of emotional problems in first grade. However, the mean scores were still in the lower range for both groups.

Compared to children of higher-educated parents ( > 5.5; short-cycle tertiary education and higher), children of lower-educated parents (< 3.60; upper secondary education and lower) had higher levels of behavioural problems across three years and higher levels of emotional problems in second grade. However, the mean scores were still in the lower range for both groups. Please see sTable 1 for specific means, standard deviations and ranges across the groups.

| **sTable 1**. Descriptive statistics of the outcome variables per household- and school-level parental education | | | | | | | | | | | |
| --- | --- | --- | --- | --- | --- | --- | --- | --- | --- | --- | --- |
|  | ***N*** | ***M*** | ***SD*** | ***Range*** |  | ***N*** | ***M*** | ***SD*** | ***Range*** |  | ***T-test*** |
|  | **Higher parental education schools** | | | |  | **Lower parental education schools** | | | |  |  |
| **Behavioural Problems** |  |  |  |  |  |  |  |  |  |  |  |
| Kindergarten | 278 | 0.71 | 0.61 | 0.0 – 2.95 |  | 148 | 0.98 | 0.75 | 0.0 – 3.34 |  | <.001^*^ |
| First Grade | 297 | 0.63 | 0.64 | 0.0 – 3.14 |  | 176 | 0.95 | 0.70 | 0.0 – 3.17 |  | <.001^*^ |
| Second Grade | 275 | 0.43 | 0.54 | 0.0 – 3.36 |  | 140 | 0.90 | 0.65 | 0.0 – 2.70 |  | <.001^*^ |
| **Emotional Problems** |  |  |  |  |  |  |  |  |  |  |  |
| Kindergarten | 275 | 0.83 | 0.59 | 0.0 – 2.87 |  | 148 | 0.85 | 0.55 | 0.0 – 3.21 |  | .716 |
| First Grade | 297 | 0.93 | 0.65 | 0.0 – 3.05 |  | 176 | 0.78 | 0.54 | 0.0 – 2.43 |  | .015^*^ |
| Second Grade | 275 | 0.87 | 0.70 | 0.0 – 3.00 |  | 140 | 0.93 | 0.66 | 0.0 – 3.25 |  | .377 |
|  | **Children of higher-educated parents** | | | |  | **Children of lower-educated parents** | | | |  |  |
| **Behavioural Problems** |  |  |  |  |  |  |  |  |  |  |  |
| Kindergarten | 205 | 0.64 | 0.56 | 0.0 – 2.95 |  | 148 | 0.96 | 0.68 | 0.0 – 2.86 |  | .001^*^ |
| First Grade | 218 | 0.66 | 0.60 | 0.0 – 2.55 |  | 183 | 0.82 | 0.66 | 0.0 – 2.90 |  | .010^*^ |
| Second Grade | 206 | 0.59 | 0.53 | 0.0 – 2.52 |  | 158 | 0.86 | 0.72 | 0.0 – 2.98 |  | <.001^*^ |
| **Emotional Problems** |  |  |  |  |  |  |  |  |  |  |  |
| Kindergarten | 203 | 0.76 | 0.58 | 0.0 – 2.87 |  | 148 | 0.84 | 0.61 | 0.0 – 3.21 |  | .230 |
| First Grade | 218 | 0.91 | 0.62 | 0.0 – 2.89 |  | 183 | 0.80 | 0.57 | 0.0 – 2.30 |  | .076 |
| Second Grade | 206 | 0.76 | 0.64 | 0.0 – 2.63 |  | 158 | 1.08 | 0.70 | 0.0 – 3.38 |  | <.001^*^ |

**Appendix B**

| **sTable 1 .** ICC, design effects, model fit indices and model building testing of multi-level modelling with random slope model versus fixed effects model | | | | | | | | | | | | | | | | | | | | |
| --- | --- | --- | --- | --- | --- | --- | --- | --- | --- | --- | --- | --- | --- | --- | --- | --- | --- | --- | --- | --- |
|  | ICC |  | Design  Effects |  | Model Fit Indices Within | | | |  |  |  | Model Fit Indices Between | | | | | |  | Fixed Effect vs Random Slope | |
|  |  |  |  |  | χ^2^ | df | RMSEA | CFI | TLI | SRMR |  | χ^2^ | df | RMSEA | CFI | TLI | SRMR |  | χ^2^ | df |
| Behavioural problems | 125 - .255 |  | 4.22 |  | 2.49 | 2 | .018 | .999 | .996 | .013 |  | 8.86^*^ | 3 | .052 | .983 | .966 | .142^*^ |  | -.16 | 1 |
| Emotional problems | .158 - .336 |  | 5.81 |  | 2.18 | 3 | .000 | 1.00 | 1.06 | .012 |  | 9.98^*^ | 2 | .074 | .920 | .760 | .214^*^ |  | 18.6^**^ | 1 |
| *Note.* ICC = intraclass correlation coefficient. Within-level and between-level fit indices were derived by saturating the latter level (6). Chi-Square Test of Model Fit, Comparative Fit Index (CFI) and Tucker Lewis Index (TLI) with critical values > .09 (7), Root Mean Square Error of Approximation (RMSEA, critical value ≤. 08) (8) and Standardized Root Mean Square Residuals (SRMR, critical value ≤ .08) (9) were used to determine model fit at both the within and between level. When cluster is small (<100), between-level SRMR values may be above the cut off value of 0.08. Therefore, Satorra Bentler Chi-Square Difference Tests were used to test the between level models to ensure that model fit at the between level was acceptable for each outcome (9). The ^*^ next to the SRMR value at the between level suggests that the results from Satorra Bentler Chi-Square Difference Tests suggest the between level models are acceptable. The model building testing of fixed effects versus random slope models were also computed by Satorra Bentler Chi-Square Difference Tests using loglikelihood (10). Significant results of the model building test indicate a need for adding a random slope parameter. For behavioural problems, we got a negative chi-square value when using the regular and the strictly positive Satorra Bentler Chi-Square Difference Tests (11). We concluded that the addition of the random slope did not improve the model fit for behavioural problems. We based our conclusion on the variance of the random slope, which was smaller than .001. ^*^*p* < .05. ^**^ *p* < .01. | | | | | | | | | | | | | | | | | | | | |

| **sTable 2.** Means and variances of the growth parameters of the unconditional multi-level latent growth models of the full sample and per GBG and the control arms | | | | | | | | | | | | | | | | |  |
| --- | --- | --- | --- | --- | --- | --- | --- | --- | --- | --- | --- | --- | --- | --- | --- | --- | --- |
|  |  | Emotional Problems | | | | | | |  | Behavioural Problems | | | | | | | |
|  |  | Mean | |  | Variance | | | |  | Mean | |  | Variance | | | | |
|  |  | **I** | **S** |  | **I _w_** | **S _w_** | **I_b_** | **S_b_** |  | **I** | **S** |  | **I _w_** | **S_w_** | **I_b_** | **S_b_** | |
| Full sample |  | .780^**^ | .135^**^ |  | .088^**^ | .022 | .049^**^ | .041^**^ |  | .760^**^ | .009 |  | .248^**^ | .003 | .053^*^ | .021^**^ | |
| GBG arm |  | .836^**^ | .065 |  | .080^*^ | .025 | .041^**^ | .028^**^ |  | .810^**^ | -.041 |  | .262^**^ | .014 | .053 | .013^**^ | |
| Control arm |  | .676^**^ | .271^**^ |  | .099^**^ | .014 | .048^*^ | .039^*^ |  | .666^**^ | .100^†^ |  | .235^**^ | .000 | .035^*^ | .023^*^ | |
| *Note.* ^†^ *p = .057*, ^*^*p* < .05, ^**^ *p* < .01. I = intercept. S = slope. w = within level. b = between level. | | | | | | | | | | | | | | | | | |

| **sTable 3.** Sensitivity analysis A: Moderation by imputed household-level parental education and school-level parental education on emotional and behavioural problems | | | | | | | | | | | | | | | |  |
| --- | --- | --- | --- | --- | --- | --- | --- | --- | --- | --- | --- | --- | --- | --- | --- | --- |
|  |  | Emotional Problems | | | | | |  | Behavioural Problems | | | | | | |  |
|  | Intercept | | |  | Slope | | |  | Intercept | | |  | Slope | | | |
|  | *B* | *S.E* | *CI(95%)* |  | *B* | *S.E* | *CI*(95%) |  | *B* | *S.E* | *CI(95%)* |  | *B* | *S.E* | *CI(95%)* | |
| **Within Level** |  |  |  |  |  |  |  |  |  |  |  |  |  |  |  | |
| Gender | .047 | .033 | -.017,.112 |  | -.029 | .032 | -.093, .034 |  | .364 | .041 | .284, .444^***^ |  | -.035 | .025 | -.084, .015 | |
| Lower parental education | .014 | .012 | -.010, .038 |  | - | - | - |  | .062 | .017 | .029, .096^***^ |  | -.008 | .007 | -.022, .006 | |
| **Between Level** |  |  |  |  |  |  |  |  |  |  |  |  |  |  |  | |
| Cluster Size | .001 | .002 | -.002, .005 |  | -.004 | .002 | -.007, .000^*^ |  | .000 | .002 | -.003, .004 |  | -.002 | .001 | -.004, .000^*^ | |
| School-level parental education | .003 | .002 | -.002, .007 |  | -.006 | .002 | -.010, -.003^**^ |  | .007 | .002 | .002, .011^**^ |  | -.001 | .002 | -.004, .003 | |
| GBG | .165 | .089 | -.010, .339 |  | -.209 | .066 | -.339, -.080^**^ |  | .136 | .089 | -.038, .310 |  | -.132 | .063 | -.255, -.009^*^ | |
| Household-level parental education -  x GBG | | - | - |  | .002 | .015 | -.027, .030 |  | - | - | - |  | - | - | - | |
| School-level parental education  x GBG | - | - | - |  | .008 | .002 | .003, .012^**^ |  | - | - | - |  | .003 | .003 | -.002, .008 | |
| *Note.* ^*^*p* < .05, ^**^*p* < .01, ^***^*p* < .001. Note that the effect of school-level parental education is small because it represents the effect at 1% change in school-level parental education. | | | | | | | | | | | | | | | | |

| **sTable 4.** Sensitivity analysis B: Moderation by complete data of household-level parental education and school-level parental education on emotional and behavioural problems | | | | | | | | | | | | | | | | |
| --- | --- | --- | --- | --- | --- | --- | --- | --- | --- | --- | --- | --- | --- | --- | --- | --- |
|  |  | Emotional Problems | | | | | | | Behavioural Problems | | | | | | | |
|  | Intercept | | |  | Slope | | |  | | Intercept | | |  | Slope | | |
|  | *B* | *S.E* | *CI(95%)* |  | *B* | *S.E* | *CI*(95%) |  | | *B* | *S.E* | *CI(95%)* |  | *B* | *S.E* | *CI(95%)* |
| **Within Level** |  |  |  |  |  |  |  |  | |  |  |  |  |  |  |  |
| Gender | .077 | .035 | .008,.146^*^ |  | -.028 | .036 | -.097, .042 |  | | .369 | .042 | .287, .451^**^ |  | -.043 | .028 | -.098, .012 |
| Lower parental education | .015 | .012 | -.008, .037 |  | - | - | - |  | | .057 | .015 | .028, .086^***^ |  | -.007 | .006 | -.019, .005 |
| **Between Level** |  |  |  |  |  |  |  |  | |  |  |  |  |  |  |  |
| Cluster Size | .001 | .002 | -.003, .005 |  | -.004 | .002 | -.007, -.001^*^ |  | | .001 | .002 | -.003, .004 |  | -.002 | .001 | -.004, .000^*^ |
| School-level parental education | .003 | .003 | -.003, .008 |  | -.006 | .002 | -.010, -.003^**^ |  | | .006 | .003 | .000, .012^*^ |  | .000 | .002 | -.004, .003 |
| GBG | .198 | .095 | .011, .385^*^ |  | -.209 | .068 | -.342, -.076^**^ |  | | .108 | .106 | -.099, .316 |  | -.101 | .061 | -.222, .019 |
| Individual-level parental education -  x GBG | | - | - |  | .005 | .017 | -.028, .038 |  | | - | - | - |  | - | - | - |
| School-level parental education  x GBG | - | - | - |  | .009 | .002 | .004, .014^***^ |  | | - | - | - |  | .003 | .003 | -.002, .008 |
| *Note.* ^*^*p* < .05, ^**^*p* < .01, ^***^*p* < .001. Note that the effect of school-level parental education is small because it represents the effect at 1% change in school-level parental education. | | | | | | | | | | | | | | | | |

**REFERENCES**

1. Ferguson CJ. An effect size primer: A guide for clinicians and researchers. In: Kazdin AE, editor. Methodological issues and strategies in clinical research. American Psychological Association; 2016. p. 301–102016.

2. Erasmus MC. Problem behavior at school interview. Rotterdam, the Netherlands: Department of Child and Adolescent Psychiatry, Erasmus MC. 2000.

3. Achenbach TM. Manual for Teacher's Report Form and 1991 Profile. Burlington: University of Vermont Department of Psychiatry1991.

4. Witvliet M, Van Lier PA, Cuijpers P, Koot HM. Change and stability in childhood clique membership, isolation from cliques, and associated child characteristics. J of Clin Child Adolesc Psychol. 2009;39(1):12-24.

5. Horoz N, Buil JM, Koot S, van Lenthe JF, Houweling AJT, Koot MH, et al. Children’s Behavioral and Emotional Problems, and Peer Relationships Across Elementary School: Associations with Individual- and School-level Parental Education. J Sch Psychol. Manuscript tentatively acceptanced.

6. Hsu H-Y, Lin JJ, Skidmore ST, Kim M. Evaluating fit indices in a multilevel latent growth curve model: A Monte Carlo study. Behav Res Methods. 2019;51(1):172-94.

7. Bentler PM, Bonett DG. Significance tests and goodness of fit in the analysis of covariance structures. Psychol Bull. 1980;88(3):588.

8. Marsh HW, Hau K-T, Wen Z. In search of golden rules: Comment on hypothesis-testing approaches to setting cutoff values for fit indexes and dangers in overgeneralizing Hu and Bentler's (1999) findings. Struct Equ Modeling. 2004;11(3):320-41.

9. Asparouhov T, Muthén B. SRMR in Mplus. Retrieved from Mplus Web Notes website: <http://www> statmodel com/download/SRMR2 pdf. 2018.

10. Satorra A, Bentler PM. A scaled difference chi-square test statistic for moment structure analysis. Psychometrika. 2001;66(4):507-14.

11. Asparouhov T, Muthén B. Computing the strictly positive Satorra-Bentler chi-square test in Mplus. Mplus Web Notes. 2010;12:1-12.
